# Actin–tropomyosin distribution in non-muscle cells

**DOI:** 10.1007/s10974-019-09514-0

**Published:** 2019-05-04

**Authors:** Dietmar J. Manstein, J. C. M. Meiring, E. C. Hardeman, Peter W. Gunning

**Affiliations:** 1 Institute for Biophysical Chemistry, OE4350, Medizinische Hochschule Hannover, 30625 Hannover, Germany; 2 Division of Structural Biochemistry, OE8830, Medizinische Hochschule Hannover, 30625 Hannover, Germany; 3 RESiST, Cluster of Excellence 2155, Medizinische Hochschule Hannover, 30625 Hannover, Germany; 4 Cellular and Genetic Medicine Unit, School of Medical Sciences, University of New South Wales, Sydney, NSW 2052 Australia

**Keywords:** Actin, Actin binding protein, Cytoplasmic protein sorting, Cytoskeleton, Myosin, Tropomyosin

## Abstract

The interactions of cytoskeletal actin filaments with myosin family motors are essential for the integrity and function of eukaryotic cells. They support a wide range of force-dependent functions. These include mechano-transduction, directed transcellular transport processes, barrier functions, cytokinesis, and cell migration. Despite the indispensable role of tropomyosins in the generation and maintenance of discrete actomyosin-based structures, the contribution of individual cytoskeletal tropomyosin isoforms to the structural and functional diversification of the actin cytoskeleton remains a work in progress. Here, we review processes that contribute to the dynamic sorting and targeted distribution of tropomyosin isoforms in the formation of discrete actomyosin-based structures in animal cells and their effects on actin-based motility and contractility.

## Introduction

Tropomyosins are a large family of integral components of most actin filaments (Gunning et al. 2005; Wang and Coluccio 2010; Meiring et al. 2018). They consist of rod-shaped dimers that polymerise along both sides of the actin filament in a head-to-tail fashion (Caspar et al. 1969; Greenfield et al. 2006). Originally isolated from muscle tissues and intensely studied for their role in muscle contraction (Bailey 1946; see Lehman and Craig 2008 and Perry 2001 for reviews), tropomyosin isoforms have since been identified in platelets and subsequently in all examined mammalian tissue types (Cohen and Cohen 1972; Garrels and Gibson 1976; Schevzov et al. 2005b; Uhlén et al. 2015) and implicated in a vast array of actin-based cytoskeletal structures (Table 1). However, details of the mechanisms underlying the sorting of cytoskeletal tropomyosin isoforms to these discrete structures remain to be elucidated.

**Table 1:** List of actin structures containing tropomyosin

Actin structure	Tropomyosin isoform	References
Sarcomeres	Tpm1.1-1.4, Tpm2.2, Tpm3.12	Jagatheesan et al. (2010)
Stress fibres	Tpm1.6, Tpm1.7, Tpm2.1, Tpm3.1/3.2, Tpm4.2	Bryce et al. (2003), Tojkander et al. (2011), Meiring et al. (2018)
Lamellipodia	Tpm1.8/1.9	Brayford et al. (2016)
Granules	Tpm3.1	Masedunskas et al. (2018)
Endosomes	Tpm3.1, Tpm4.2	Gormal et al. (2017)
Cell cortex	Tpm1.9, Tpm3.1/3.2, Tpm4.2	Kee et al. (2015, 2017, 2018), Sung et al. (2000), Martin et al. (2010)
Epithelial zonula adherens	Tpm3.1/3.2, Tpm4.2	Caldwell et al. (2014), Meiring et al. (2018)
Podosomes	Tpm1.8/1.9, Tpm4.2	McMichael et al. (2006)
Cleavage furrow	Tm1A^a^, Tpm1.6/1.7/2.1, Tpm3.1	Goins and Mullins (2015), Hughes et al. (2003)
Mitotic spindles	Tm1J^a^	Goins and Mullins (2015)
Golgi-associated short filaments	Tm1J^a^, Tm2A^a^, Tpm3.2	Goins and Mullins (2015), Percival et al. (2004)
Filopodia	Tpm1.7	Creed et al. (2011)
Post-synaptic density of dendritic spines	Tpm3.1-3.9, Tpm4.2	Suchowerska et al. (2017), Had et al. (1994)
Sarcoplasmic reticulum	Tpm4.2	Vlahovich et al. (2009)
Endoplasmic reticulum	Tpm4.2	Kee et al. (2017)
Skeletal muscle triad	Tpm3.1/3.2, Tpm4.2	Vlahovich et al. (2009)

^a^*Drosophila* tropomyosin isoforms

Protein sorting has been extensively studied over the last 50 years. The two primary mechanisms involve either sorting at the level of individual proteins or within vesicles. In general, sorting involves recognition of a signal sequence within the protein. Signal sequences can usually be experimentally transferred to a marker protein to demonstrate the autonomous function of the signal sequence (see Alberts et al. 2015). Such signal sequences cannot be detected in tropomyosins, which makes their sorting particularly interesting (Martin et al. 2010).

Cytoskeletal tropomyosin isoforms are thought to determine actin filament function by influencing the stability of actin filaments, promoting or inhibiting the binding of other actin binding proteins, and regulating the activity of myosin isoforms in an isoform dependent manner (Gunning et al. 2005; Gunning et al. 2015) (Table 2). This is apparent from studies where individual tropomyosin isoforms have been over-expressed resulting in a change in the predominant actin structures, a change in the ratio of monomeric to polymeric actin, and altered recruitment of actin binding proteins (Bryce et al. 2003; Jalilian et al. 2015; Creed et al. 2011; Bach et al. 2009). Knock down of subsets of tropomyosin isoforms in cultured cells results in a reduction or loss of specific actin structures including cell–cell junctions (Caldwell et al. 2014); and stress fibres (Tojkander et al. 2011) or expansion of the lamellipodium (Brayford et al. 2016). Furthermore, isoform-specific effects on the stability of actin–tropomyosin interactions and ability to resist cofilin severing (Gateva et al. 2017) and myosin activity have been reproduced in cell-free assays using purified actin, tropomyosin isoforms, cofilin, alpha-actinin and nonmuscle myosin-2B (NM-2B) (Pathan-Chhatbar et al. 2018). Moreover, cytoskeletal tropomyosins have been reported to protect actin filaments from severing by gelsolin in an isoform-dependent manner in cell-free assays (Kis-Bicskei et al. 2018; Khaitlina et al. 2013).

**Table 2:** List of isoform specific functions of tropomyosin

Tropomyosin isoform	Function	References
Tpm1.6	Stabilizes stress fibres	Tojkander et al. (2011)
	Modulates myosin-1b and myosin-1c actin-affinity and motor activity	Tang and Ostap (2001), and Kee et al. (2015)
	Rescues transformed cells	Gimona et al. (1996)
	Protects filaments against severing by cofilin	Gateva et al. (2017)
Tpm1.7	Promotes formation of filopodia	Creed et al. (2011)
	Co-operatively associates with actin filaments with fascin	Creed et al. (2011)
	Recruits ADF	Creed et al. (2011)
	Protects filaments against severing by cofilin	Gateva et al. (2017)
	Inhibits neuronal morphogenesis	Schevzov et al. (2005a)
Tpm1.8/1.9	Promotes focal adhesion formation and lamellipodial persistence	Brayford et al. (2016)
	Regulates Cystic Fibrosis Transmembrane conductance Regulator (CFTR) in the membrane	Dalby-Payne et al. (2003)
	Regulates mammary gland differentiation	Zucchi et al. (2001)
Tpm2.1	Helps to establish focal adhesions	Tojkander et al. (2011, 2012), Desouza-Armstrong et al. (2017)
	Restores stress fibres in transformed cells	Prasad et al. (1993), Desouza-Armstrong et al. (2017)
	Sensitizes cells to apoptosis and anoikis	Desouza-Armstrong et al. (2017), Raval et al. (2003)
	Tension sensing	Wolfenson et al. (2016)
Tpm3.1/3.2	Regulates insulin-stimulated GLUT4 transport to the plasma membrane and glucose uptake	Kee et al. (2015, 2017)
	Regulates cell motility and migration	Bach et al. (2009)
	Promotes formation of NM-2A enriched stress fibres	Bryce et al. (2003)
	Activates NM-2A ATPase	Gateva et al. (2017)
	Promotes cell proliferation	Schevzov et al. (2015)
	Supports myosin-5a engagement and activity	Sckolnick et al. (2016)
Tpm4.2	Recruits NM-2A to stress fibres	Tojkander et al. (2011, 2012)
	Activates NM-2A ATPase	Gateva et al. (2017)
	Supports ER to Golgi trafficking	Kee et al. (2017)
	Inhibits formation of filopodia	Tojkander et al. (2011)

## Tropomyosin structure and dynamics

**Fig. 1:**
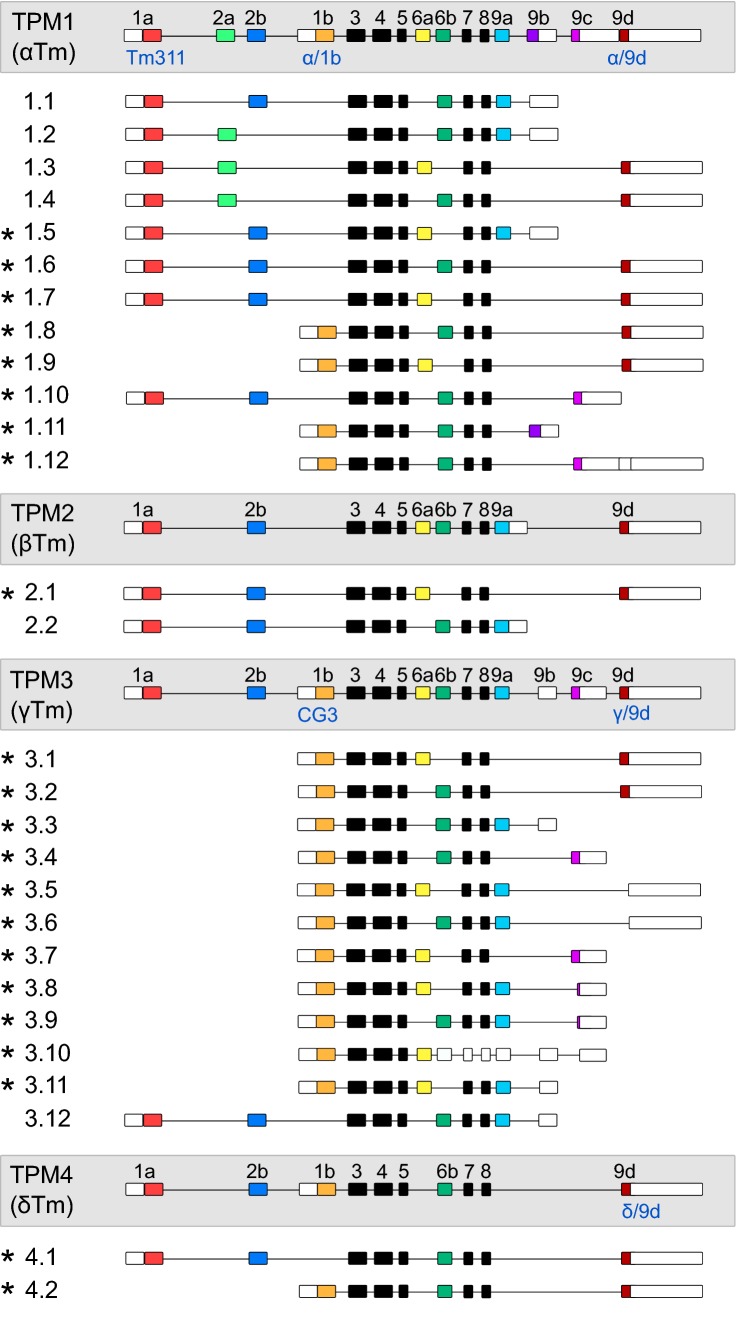
Schematic of mammalian tropomyosin genes and known isoforms generated as a result of alternative exon splicing. Coloured boxes represent alternately spliced protein coding exons, black boxes indicate protein coding exons common to all isoforms, lines represent introns and white boxes represent untranslated regions. Commercially available antibodies are noted in blue text underneath their respective epitopes. Isoforms marked with an asterix (*) are cytoplasmic. (Color figure online). Figure adapted from Schevzov et al. (2011). Note that there is evidence for additional isoforms

Mammalian cells have four different tropomyosin genes that give rise to at least 28 different isoforms as a result of alternate splicing (see Fig. 1 for known isoforms) (Schevzov et al. 2011). Tropomyosin dimers span across 6–7 subunits of actin depending on whether the isoform is a low molecular weight (LMW) or a high molecular weight (HMW) isoform (Barua et al. 2011). The structural difference between HMW and LMW isoforms is that HMW isoforms contain exon 1a and either 2a or 2b derived sequences, while LMW isoforms contain sequences derived from exon 1b, but none from exon 2 (Fig. 1) (Wieczorek et al. 1988, Schevzov et al. 2011). Due to exon sharing between tropomyosin isoforms, antibodies or reagents developed against tropomyosins typically target more than one tropomyosin isoform (Schevzov et al. 2011). As a result certain tropomyosin isoforms are not easily distinguishable. In these cases tropomyosin isoforms are addressed as subsets, such as Tpm3.1/3.2 or Tpm1.8/1.9.

The C-terminal domain of tropomyosin can bind the N-terminus of an adjacent tropomyosin and this interaction is important for stabilizing tropomyosin on actin filaments (Caspar et al. 1969, see Tobacman 2008 for review). End-to-end linked tropomyosin associates with approximately 1000-fold greater affinity than an individual tropomyosin dimer, thus promoting the complete gap-free coating of actin filaments (Wegner, 1980). While tropomyosins are known to be able to coat unbranched actin filaments, an in vitro study found that *Drosophila* non-muscle tropomyosin 1A (for which there is no known vertebrate counterpart isoform) was also unable to bind Arp2/3 nucleated branched rabbit muscle actin networks after severing of the branches with cofilin (Hsiao et al. 2015). This and the results of a similar study, which used rabbit muscle tropomyosins, suggest that a tropomyosin polymer is first nucleated at the minus-end of the actin filament before extending towards the plus-end (Hsiao et al. 2015; Bugyi et al. 2010). In branched networks the Arp2/3 complex blocks the minus-end of the actin filament and this is thought to be the reason why tropomyosin cannot bind to branched filaments (Hsiao et al. 2015). In agreement with the in vitro data, most tropomyosin isoforms are absent from lamellipodia, the largest hub of branched actin networks in the cell. However, a recent study showed selective recruitment of Tpm1.8/1.9 to lamellipodia (Brayford et al. 2016). Tpm1.8/1.9 recruitment to lamellipodia can be perturbed by depletion of either coronin 1B or cofilin, suggesting that coronin 1B- and cofilin-mediated actin debranching must occur before Tpm1.8/1.9 is able to associate with the Arp2/3 nucleated actin filaments.

Fluorescently-tagged tropomyosin Tpm3.1 has been observed to cycle on and off actin filaments *in cellulo* and in vivo in rodents, suggesting that tropomyosins themselves are more dynamic than actin filaments (Appaduray et al. 2016). In vitro FRAP experiments report that the dynamics of HMW tropomyosins are slower than those of the LMW isoforms, suggesting that HMW isoforms associate in a more stable fashion with actin filaments (Gateva et al. 2017).

Another factor found to impact tropomyosin affinity for actin is N-terminal acetylation. Based on the fact that 80% of human proteins are acetylated at their N-terminus (Arnesen et al. 2009; Silva and Martinho 2015), it is likely that cytoskeletal tropomyosins too are predominantly acetylated and this has been confirmed for Tpm3.1/3.2 and Tpm4.2 (Meiring et al. 2018). However, the extent to which all individual cytoskeletal tropomyosin isoforms are acetylated remains unknown. The introduction of an acetyl group at the N-terminus of a protein eliminates the positive charge of the N-terminal amino group. The in vitro actin-binding capacities of skeletal muscle as well as smooth muscle tropomyosin isoforms are greatly enhanced by N-terminal acetylation, which strengthens head-to-tail Tpm–Tpm contacts. In fact, both isoforms have a very poor actin binding capacity without N-terminal modification (Urbancikova and Hitchcock-DeGregori 1994; Coulton et al. 2006). Sarcomeric tropomyosin isoforms are known to be acetylated in vivo and N-acetylation of their N-terminal methionine contributes critically to the formation and stabilization of a stable overlap complex (Frye et al. 2010; Lehman et al. 2014). In contrast, the ability of cytoskeletal tropomyosin isoforms to interact with F-actin was reported to be less critically affected by N-terminal acetylation. Other subtle changes near the N-terminus were reported to have a critical effect on tropomyosin head-to-tail interactions including a compensatory effect in regard to the N-terminal acetylation of sarcomeric tropomyosin isoforms. The ability of sarcomeric tropomyosin isoforms to polymerize and efficiently bind to F-actin was shown to be enhanced by the addition of a single glycine residue, an Ala–Ser dipeptide, or a Gly–Ala–Ser tripeptide to their N-termini (Frye et al. 2010; Greenfield et al. 2001; Monteiro et al. 1994). Similar, the effect of a lack of N-acetylation of the skeletal muscle isoform Tpm1.1 on actin binding was compensated by the replacement of exons 1a and 2b with the N-terminal exon 1b that is present in cytoskeletal isoforms Tpm1.8, Tpm1.12, or Tpm3.1 (Moraczewska et al. 1999). Exon 9-encoded sequences were reported to correspond to another decisive determinant in regard to head-to-tail interactions. Replacement of the striated muscle-specific exon 9a encoded C-terminus with exon 9d, which is found in smooth muscle and cytoskeletal tropomyosin isoforms, allowed nonacetylated hybrid tropomyosin to efficiently bind to F-actin (Cho and Hitchcock-DeGregori 1991).

## Tropomyosin expression and turnover

Given the diversity of functions of different tropomyosin isoforms and their association with discrete cytoskeletal structures, it follows that their cellular concentration and localisation needs to be tightly regulated. Indeed, when fibroblasts were synchronised via serum starvation, all tropomyosin isoforms tested showed cell cycle-dependent changes in protein levels. In addition, some isoforms showed changing localisation patterns (Percival et al. 2000). This indicates that tropomyosin isoform concentration and distribution are regulated both spatially and temporally. For example, Tpm3.1/3.2 expression is decreased during cell migration and increased during cell reattachment (Percival et al. 2000; Bach et al. 2009; Lees et al. 2013). This is in agreement with the finding that Tpm3.1 stabilises focal adhesions and reduces the speed of cell migration (Bach et al. 2009). Tpm3.2 has been reported to have a similar function to Tpm3.1 (Caldwell et al. 2014).

Total muscle tropomyosin levels are known to be tightly regulated for the sarcomere-associated tropomyosin isoforms Tpm1.1, Tpm2.2, and Tpm3.12 (Schevzov and O’Neill 2008). The over-expression of muscle Tpm2.2 in adult mouse heart was observed to cause depletion of endogenous muscle tropomyosins from the α-tropomyosin gene (Muthuchamy et al. 1995). Similarly, overexpression of a mutant Tpm3.12 in mouse muscle resulted in a reduction of endogenous Tpm2.2 and to a lesser degree Tpm1.1 (Corbett et al. 2005). In the case of cytoskeletal tropomyosins, isoform compensation mechanisms have only been observed in red blood cells. Here, the targeted deletion in alternatively spliced exon 9d of Tpm3 (Tpm3/9d(−/−)) leads to absence of Tpm3.1 along with a compensatory increase in Tpm1.9 of sufficient magnitude to maintain normal total tropomyosin content (Sui et al. 2017). In contrast, no such feedback mechanism has been observed for the cytosolic tropomyosins produced by mouse embryo fibroblast, primary hippocampal neurons, and mouse eye lenses. In these cell types neither the overexpression (Schevzov et al. 2008) nor the knockdown (Cheng et al. 2018) of a cytosolic tropomyosin isoform results in compensating changes in the production of other isoforms. In the case of Tpm3.1, overexpression leads to an increase in F-actin (Schevzov et al. 2008; Jalilian et al. 2015; Kee et al. 2015).

To maintain protein homeostasis, cells actively maintain a delicate equilibrium between protein degradation and protein synthesis. The associated turnover of tropomyosin isoforms has been studied by subjecting cells to a pulse with radio-labelled methionine, and collecting cell lysates at several time points afterwards. Newly synthesised proteins directly after the pulse will contain the largest fraction of radio-labelled methionine. Therefore, the rate of decrease in radioactive protein with time may be interpreted as the rate of protein turnover. In this way the major HMW tropomyosin isoforms identified (Tpm1.6, 1.7 and 2.1) were discovered to turnover with a half-life of 1.5–8.25 h. The LMW isoforms identified (Tpm3.1/3.2, Tpm4.2) showed minimal turnover for the duration of the experiment (half-life > 20 h) (Lin et al. 2008). This may reflect the proteasomal turnover of HMW, but not LMW tropomyosins upon their dissociation from actin filaments (Meiring et al. 2018).

## Tropomyosin recruitment

While differences are observed in localisation between tropomyosin isoforms (Table 1), it is still not clear what determines the recruitment of different isoforms to specific actin structures (Tojkander et al. 2011; Martin and Gunning 2008; Suchowerska et al. 2017; Brayford et al. 2016). There is evidence that tropomyosin is not actively transported to particular locations in the cell (Martin et al. 2010). The localisation of cytoskeletal tropomyosin isoforms can be perturbed via drugs that target actin assembly such as Cytochalasin D (Dalby-Payne et al. 2003; Schevzov et al. 1997) and targeting is abolished in mutated Tpm3.1 which is made incapable of assembling into a co-polymer with actin (Martin et al. 2010). It is therefore likely that cytoskeletal tropomyosin sorting requires incorporation of the tropomyosin into a co-polymer with actin.

Currently, one hypothesis for selective tropomyosin recruitment is that formin nucleators preferentially promote the formation of co-filaments of β- or γ-actin filaments with particular cytoskeletal tropomyosin isoforms. This hypothesis is supported by experiments performed in yeast. The fission yeast *Schizosaccharomyces pombe* produces acetylated and un-acetylated variants of a single tropomyosin isoform (Cdc8). Formation of actin co-filaments with the acetylated and un-acetylated CDC8 variants result in distinct functions. In this system Johnson et al. (2014) showed that swapping the localisation of two formin homologues, For3 and Cdc12, resulted in a switch in the localisation of N-terminally acetylated and un-acetylated Cdc8. A related study performed with human osteosarcoma cells showed that the formin mDia2 is able to influence tropomyosin recruitment (Tojkander et al. 2011). However, the extent to which this effect is isoform-specific remained unclear. A recent study of actin assembly on salivary granules in live mice supports the view that actin and Tpm3.1 co-assemble during their recruitment to granules, whilst the recruitment of non-muscle myosin-2A (NM-2A) to granules occurs only after a significant delay (Masedunskas et al. 2018). Based on the observation that cytoskeletal tropomyosin isoforms co-assemble with actin, the notion that formin nucleators selectively produce actin–tropomyosin co-polymers is indeed plausible.

The formin-mediated tropomyosin recruitment model proposed in Johnson et al. (2014) suggests that tropomyosins are recruited via a mechanism that involves the N-terminus of tropomyosin directly interacting with a formin. This model was derived from the observation that the *S. pombe* tropomyosin CDC8 is specifically sorted based on its N-terminal acetylation status and predicts that the N-termini of tropomyosin isoforms determine isoform localisation (Johnson et al. 2014). However, mammalian tropomyosin chimeras with N-terminal exons from differently localising tropomyosins do not show altered tropomyosin localisation, indicating that tropomyosin localisation is not dependent on the N-terminal region in mammals (Martin et al. 2010). Structural biochemical studies have suggested that N-terminal acetylation of tropomyosin also impacts tropomyosin structure distant from the N-terminus (Johnson et al. 2017; East et al. 2011). Perhaps then, rather than the N-terminus of tropomyosin associating with a formin directly, different formin isoforms may produce actin filaments with different intrinsic physical properties that are more favourable for certain tropomyosins or actin binding proteins. Although controversial, conventional and cryo-electron microscopy has revealed that F-actin has several possible states and conformations (Galkin et al. 2010; Egelman and Orlova 1995; von der Ecken et al. 2015). Further, the formin mDia1 is able to nucleate cofilin-resistant actin filaments by rotating along the axis of a tethered actin filament during elongation and twisting the filament (Mizuno et al. 2018). One potential mechanism for formin-mediated actin filament specialisation may then be to impart a different helical rotation or conformational state on an actin filament (Papp et al. 2006), which may in turn be more conducive to binding by different tropomyosin isoforms. However, a recent test of the roles of two formins, either separately or in combination, to determine the assembly of tropomyosin isoforms into co-polymers with actin failed to identify any impact in mammalian cells ruling out a simple one formin for one tropomyosin isoform relationship (Meiring et al. 2019).

It is likely that there is more than one mechanism in place for the assembly of actin and tropomyosin co-polymers, since actin nucleated by Arp2/3 may be bound by tropomyosin after debranching (Brayford et al. 2016). Moreover, recent studies have demonstrated that actin nucleated by Arp2/3 at the cell membrane is remodelled into various other types of tropomyosin-containing structures such as focal adhesions, stress fibres and the contractile actin ring at the zonula adherens (ZA) (Brayford et al. 2016; Tojkander et al. 2015; Michael et al. 2016).

Complexes formed by actin (A), myosin (M) and tropomyosin (Tpm) isoforms contain large stereospecific contact areas. Thus, the A–M, M–Tpm, and A–Tpm contact areas comprise 1800, 300, and 210 Å^2^, respectively (Behrmann et al. 2012; von der Ecken et al. 2016). Based on atomic models of discrete A-Tpm-M complexes, it is possible to begin relating known differences in their interactions to the structural features of individual myosin and tropomyosin isoforms (Manstein and Mulvihill 2016). The structure of a human actomyosin–tropomyosin complex, composed of the motor domain of NM-2C, filamentous γ-actin and Tpm3.1 shows that the interface between the NM-2C motor domain and F-actin is formed on the myosin-side mainly by contacts between the helix-loop–helix motif, the CM-loop, loop 2, loop 3, and the proline-rich ‘activation’ loop and on the actin-side by subdomains 1 and 3 of one actin molecule and the D-loop in subdomain 2 of the adjacent actin molecule. Arginine 384 in loop 4 of NM-2C contributes a critical interaction with a cluster of acidic residues on the Tpm3.1 surface (von der Ecken et al. 2016). Therefore, the contributions of differences in the length and amino acid composition of loop 4 and the impact of variations in the periodic pattern of evolutionarily conserved basic and acidic residues on the tropomyosin surface are now starting to emerge. They explain why actin–tropomyosin co-filaments, which contain different cytoplasmic tropomyosin isoforms, show distinct preferential interactions with myosins in functional assays (Stark et al. 2010, Clayton et al. 2014, 2015, Hundt et al. 2016, Kee et al. 2015, von der Ecken et al. 2016, Gateva et al. 2017). Thus, Tpm3.1- and Tpm4.2-containing actin filaments were reported to enhance the recruitment of NM-2A and NM-2B, respectively (Hundt et al. 2016; Pathan-Chhatbar et al. 2018). Tpm4.2 and Tpm1.8 were shown to promote the processive behaviour of NM-2A and NM-2B, respectively (Hundt et al. 2016; Pathan-Chhatbar et al. 2018). In the case of Myo1c, it was reported that Tpm3.1-containing actin filaments have a restricting effect on actin binding (Kee et al. 2015). Tpm1.12 was shown to weaken the recruitment of NM-2B significantly (Pathan-Chhatbar et al. 2018).

On the one hand, cytoskeletal tropomyosin isoforms could be acting as a filter, controlling the recruitment of specific myosin isoforms to actin–tropomyosin co-filaments. On the other hand, the contact of a cluster of a specific type of myosin with an actin–tropomyosin co-filament could be driving the exchange of the associated tropomyosin isoform for one that displays greater stereospecific interactions. There exists currently no strong evidence that favours one process over the other. Both scenarios are in broad agreement with the available in vitro data, but require more direct experimental verification. In addition, it was shown that the exchange of tropomyosin isoform can affect the coupling between actin binding and nucleotide turnover within the myosin motor. Tpm3.1 was reported to inhibit the ability of Myo1c to enter into a force-generating state (Kee et al. 2015). In the case of NM-2B, the presence of saturating concentrations of Tpm1.8, Tpm1.12, or Tpm3.1 was reported to affect the release of the ATP hydrolysis products ADP and phosphate from the active site to different extents (Pathan-Chhatbar et al. 2018). As phosphate release gates a transition from weak to strong F-actin–binding states and ADP release has the opposite effect, both the affinity for filamentous actin in the presence of ATP and the duty ratio, the fraction of time that NM-2B spends in strongly F-actin bound states during ATP turnover, are affected. Compared to bare F-actin, the duty ratio is thereby increased threefold in the presence of saturating concentrations of Tpm1.12 and fivefold for both Tpm1.8 and Tpm3.1. The presence of Tpm1.12 extends the time required per ATP hydrolysis cycle 3.7-fold, whereas it is shortened by 27 and 63% in the presence of Tpm1.8 and Tpm3.1, respectively. During active turnover of ATP, the affinity for F-actin was reported to be significantly increased by all three Tpm isoforms. The apparent second-order rate constant *k*_cat_/*K*_app-actin_, which reflects the behaviour of the fully activated complex and is a measure of the coupling efficiency between the actin- and nucleotide-binding sites of myosin (Dürrwang et al. 2006), was reported to increase 2.9-fold in the presence of Tpm1.8 and 2.5-fold in the presence of Tpm3.1 while a 14.3% reduction was observed in the presence of Tpm1.12 (Pathan-Chhatbar et al. 2018). The exchange of isoforms can thus gear motor activity towards slower or faster movement, tension holding or active constriction (Hundt et al. 2016; Gateva et al. 2017; Pathan-Chhatbar et al. 2018). The associated tropomyosin isoform-specific changes in the frequency, duration, and efficiency of actomyosin interactions establish a molecular basis for the ability of these complexes to support cellular processes with divergent demands in regard to speed, force, and processivity.

## Tropomyosin association with stress fibres

Actin structures may be bundled or cross-linked in cells for the purpose of building stronger structures or structures capable of exerting force or tension. Several specific actin bundling and cross-linking proteins exist. Fascin, fimbrin and α-actinin all cross-link actin filaments into bundles of parallel filaments. However, they each have different roles and localisations (Adams 1995; Kovac et al. 2013; Yamashiro et al. 1998; Bretscher 1981). Fascin and fimbrin are best known for their role in the formation of membrane protrusions (Yamashiro et al. 1998; Bretscher 1981). Meanwhile α-actinin is known to be a critical cross-linker in stress fibres (Kovac et al. 2013). Other actin cross-linkers found in stress fibres include filamin (Wang et al. 1975) and palladin (Dixon et al. 2008). However, their exact contributions in stress fibres have not yet been determined. Finally, it should be noted that single- and double-headed cytoskeletal myosin isoforms can also support the dynamic cross-linking of actin filaments (Laevsky and Knecht 2003).

Currently, the only structures known to recruit all the major non-muscle and non-neuronal tropomyosins are stress fibres (Tojkander et al. 2011). Ventral stress fibres are contractile structures that allow cells to respond to mechanical force, to remodel connective tissue, apply force on neighbouring cells or on tubules and ducts (Pellegrin and Mellor 2007). Stress fibres in non-motile cells tend to be thick and relatively stable, while stress fibres in motile cells are typically thin and more dynamic (Pellegrin and Mellor 2007). Moreover, the structure of the stress fibre depends on the stress fibre type. Dorsal stress fibres are not contractile. They contain α-actinin and Tpm1.6, but not NM-2 isoforms (Tojkander et al. 2011). Ventral stress fibres, transverse arcs and the perinuclear actin cap are all contractile structures with a quasi-sarcomeric organisation consisting of actin filaments in a bipolar arrangement, crosslinked by α-actinin and NM-2 (Tojkander et al. 2012). Contractile stress fibres share structural similarities with other contractile actin structures. These include the contractile actin ring that pinches the cell membrane in cytokinesis (Henson et al. 2017) and the contractile actin ring that supports cell–cell adhesions in epithelial cells (Ebrahim et al. 2013). The major non-muscle tropomyosins have been found in contractile stress fibres (Tpm1.6, 1.7, 2.1, 3.1/3.2 4.2). Depletion of any of these isoforms was found to perturb the stress fibre network (Tojkander et al. 2011). This suggests that the network is formed by different types of actin filaments, whose functional properties are defined by the association with different tropomyosins. However, it is not yet known how the different isoforms are organised with respect to one another or to the other actin binding proteins found in stress fibres. Of the major tropomyosin isoforms present, Tpm4.2 depletion impaired NM-2 recruitment, but had only a minor impact on stress fibre integrity. The specific functions of other stress fibre-associated isoforms are not fully understood (Tojkander et al. 2011).

Overexpression of Tpm3.1 leads to enrichment of non-muscle myosin isoform NM-2A, but not NM-2B or NM-2C in stress fibres (Bryce et al. 2003). The myosin ATPase activity and sliding velocity in complex with Tpm3.1-decorated F-actin is enhanced for non-muscle myosin NM-2A and 2C, but not for 2B (Barua et al. 2014). Tpm4.2 decoration of actin filaments accelerates the NM-2A ATPase activity, specifically targeting the rate limiting step of phosphate release in cell-free assays. Moreover, Tpm4.2-decorated filaments induce a transition towards an increased processive behaviour under resisting force (Hundt et al. 2016). Meanwhile NM-2B catalytic activity is increased in the presence of Tpm1.8 and Tpm3.1, but decreased in the presence of Tpm1.12 (Pathan-Chhatbar et al. 2018). Tpm3.1 and Tpm4.2 further show rapid cycling on-and-off actin filaments and fail to protect actin filaments from disassembly (Appaduray et al. 2016, Gateva et al. 2017). Together these data suggest a role for Tpm3.1 and Tpm4.2 in regulating NM-2 function in stress fibres, but not actin filament stabilisation.

Along with NM-2 isoforms, actin and α-actinin, Tpm2.1 have been implicated in the rigidity sensing of contractile units at the cell periphery (Wolfenson et al. 2016). The HMW Tpm1.6 shows stable interactions with F-actin and protects filaments against cofilin-mediated disassembly, but does not activate NM-2A (Gateva et al. 2017). These results support the view that different types of actin–tropomyosin co-filaments have different preferential interactions with myosins and make different functional contributions within stress fibres. However, further work is required to elucidate the organisation and precise contributions of the different tropomyosin isoforms within stress fibres.

## Conclusions and prospects

The cytoskeletal tropomyosins have proved to be an elegant evolutionary solution to the complex problem of the regulation of cell architecture. By virtue of their co-polymerisation with actin (Masedunskas et al. 2018), they are responsible for the generation of multiple distinct filaments with their own functional characteristics. With few exceptions, all animal cells contain multiple types of specialised actin–tropomyosin co-filaments with associated functional diversity. The mechanism by which these specialised filaments are generated remains a challenge. The simplest mechanism for most filaments is co-polymerisation of actin and tropomyosin. The tropomyosin isoforms appear to have an intrinsic preference to form homo-polymers (Gateva et al. 2017), which focusses the problem on what determines the initial selection of a tropomyosin isoform to initiate co-polymerisation with actin. Is it the actin filament nucleator that initiates the actin filament or the availability and/or activity of local actin binding proteins and myosin motors at the site of assembly? Whatever the mechanism, we can be confident that it will be highly responsive to the local requirements of the cell. The importance of elucidating the assembly mechanism is that it will provide an understanding of how multiple distinct filaments can be assembled into higher order structures such as stress fibres. Moreover, elucidation of the assembly mechanism promises to provide a direct link between local trigger signals and isoform assembly choices. The resulting knowledge will help to link the formation of specific sets of actin–tropomyosin co-filaments and the associated local activity of a well-defined subset of actin binding proteins and myosin motors to the particular functional demands invoked by the trigger signal.
